# Synaptotagmins interact with APP and promote Aβ generation

**DOI:** 10.1186/s13024-015-0028-5

**Published:** 2015-07-23

**Authors:** Vivek Gautam, Carla D’Avanzo, Oksana Berezovska, Rudolph E. Tanzi, Dora M. Kovacs

**Affiliations:** Genetics and Aging Research Unit, MassGeneral Institute for Neurodegenerative Disease, Massachusetts General Hospital, Harvard Medical School, Charlestown, MA 02129 USA; MassGeneral Institute for Neurodegenerative Disease, Massachusetts General Hospital, Harvard Medical School, Charlestown, MA 02129 USA

**Keywords:** Alzheimer’s disease, APP, BACE1, Aβ, APP-interacting proteins, Synaptotagmin, Synaptic vesicles, Proteomics

## Abstract

**Background:**

Accumulation of the β-amyloid peptide (Aβ) is a major pathological hallmark of Alzheimer’s disease (AD). Recent studies have shown that synaptic Aβ toxicity may directly impair synaptic function. However, proteins regulating Aβ generation at the synapse have not been characterized. Here, we sought to identify synaptic proteins that interact with the extracellular domain of APP and regulate Aβ generation.

**Results:**

Affinity purification-coupled mass spectrometry identified members of the Synaptotagmin (Syt) family as novel interacting proteins with the APP ectodomain in mouse brains. Syt-1, −2 and −9 interacted with APP in cells and in mouse brains *in vivo*. Using a GST pull-down approach, we have further demonstrated that the Syt interaction site lies in the 108 amino acids linker region between the E1 and KPI domains of APP. Stable overexpression of Syt-1 or Syt-9 with APP in CHO and rat pheochromocytoma cells (PC12) significantly increased APP-CTF and sAPP levels, with a 2 to 3 fold increase in secreted Aβ levels in PC12 cells. Moreover, using a stable knockdown approach to reduce the expression of endogenous Syt-1 in PC12 cells, we have observed a ~ 50 % reduction in secreted Aβ generation. APP processing also decreased in these cells, shown by lower CTF levels. Lentiviral-mediated knock down of endogenous Syt-1 in mouse primary neurons also led to a significant reduction in both Aβ40 and Aβ42 generation. As secreted sAPPβ levels were significantly reduced in PC12 cells lacking Syt-1 expression, our results suggest that Syt-1 regulates Aβ generation by modulating BACE1-mediated cleavage of APP.

**Conclusion:**

Altogether, our data identify the synaptic vesicle proteins Syt-1 and 9 as novel APP-interacting proteins that promote Aβ generation and thus may play an important role in the pathogenesis of AD.

**Electronic supplementary material:**

The online version of this article (doi:10.1186/s13024-015-0028-5) contains supplementary material, which is available to authorized users.

## Introduction

Alzheimer’s disease (AD) is one of the most common debilitating neurodegenerative diseases [1]. The neuropathological hallmarks of AD primarily derive from aggregation and deposition of misfolded proteins, in particular the β-amyloid (Aβ) peptide that oligomerizes to form extracellular senile plaques [2] and the hyperphosphorylated tau protein that aggregates into neurofibrillary tangles [3, 4]. Numerous studies have shown that AD symptoms directly correlate with the amount of pathogenic oligomeric Aβ in the brain [5, 6]. Aβ is generated from the Amyloid Precursor Protein (APP), a type I membrane protein [5, 7]. APP can undergo either non-amyloidogenic or amyloidogenic processing depending on the secretases that cleave the protein. In the non-amyloidogenic cascade, APP is sequentially cleaved by α-secretase and γ-secretase thus generating p3 and AICD fragments [8]. In the amyloidogenic processing, BACE1 first generates βCTF that is further processed by the γ-secretase complex to produce pathogenic Aβ peptides and AICD fragments [9–15]. Aβ peptides oligomerize and aggregate in the form of plaques, resulting in inflammation and neuronal cell death [16, 17].

APP is a ubiquitously expressed ~105 kDa transmembrane glycoprotein [5]. In the brain, APP is found at both presynaptic and postsynaptic terminals and plays an important role in various neuronal functions such as synapse formation, neuronal migration, neurite outgrowth, synaptic plasticity, synaptic transmission and learning and memory [18–21]. APP is known to traffic axonally by interacting with the kinesin light chain and localize at the presynaptic terminal [22–25]. There, it undergoes proteolytic processing and releases Aβ at the synapse [25–27]. Although numerous APP-interacting proteins have been identified [28, 29], not much is known about synaptic proteins that interact with the ectodomain of APP and may regulate Aβ generation.

The Synaptotagmin (Syt) family of proteins is known to regulate membrane trafficking and fusion [30, 31]. Syts are type-I transmembrane proteins with a short amino-terminal domain and a large carboxyl-terminal cytoplasmic region harboring the Ca^2+^-binding domains C2A and C2B (Additional file 1: Figure S1B) [30, 32, 33]. Importantly, Syt-1, −2 and −9 are known to act as calcium sensors on synaptic vesicles and play a major role in synaptic vesicle membrane fusion events [34]. Recent studies have also shown that APP is present in purified synaptic vesicle preparations along with its secretases and Aβ [35, 36].

Here, we performed an unbiased MS-coupled affinity chromatography screen to search for synaptic proteins that interact with the extracellular domain of APP. Our study identifies Syt-1, −2 and −9 as novel APP-ectodomain binding proteins and demonstrates that Syt-1 is a physiological interactor of APP. Moreover, we show that both Syt-1 and Syt-9 increase Aβ levels likely via BACE1-mediated APP processing and thus may play an important role in Alzheimer’s disease pathology. Overall, our studies reveal an important novel function for Syt family of synaptic vesicle proteins in regulating Aβ generation.

## Materials and methods

### Antibodies and reagents

Rabbit anti-APP antibody and anti-sAPPβ antibodies were described previously [37]. Syt-1 mouse monoclonal and Syt-5/9 rabbit polyclonal antibodies were obtained from Synaptic Systems (Goettingen, Germany). Mouse monoclonal anti-GAPDH antibody was purchased from Cell Signaling (Danvers, MA) while mouse anti-V5 antibody was from Life Technologies (Grand Island, NY). Anti-Syt-1 mouse monoclonal antibody (ASV30) and Isopropyl-beta-D-thiogalactopyranoside (IPTG) were from Thermo Fisher Scientific (Waltham, MA).

### Plasmids construction

To generate NH_2_-terminal GST-tagged APP ectodomain constructs, different ectodomain regions of APP were first amplified with the help of polymerase chain reaction (PCR) using the following forward and reverse primers, E1 domain: forward, ATATATGTCGACTCTGGAGGTACCCACTGATGG; reverse, TATATAGCGGCCGCCTAAGCCAGTGGGCAACACAC; E1+ KPI domain: forward, ATATATGTCGACTCTGGAGGTACCCACTGATGG; reverse, TATATAGCGGCCGCCTAGGCATCAGGGGTACTGGC; E2 domain: forward, ATATATGTCGACTGCAGCCAGTACCCCTGATG; reverse, TATATAGCGGCCGCCTAGGCCAAGACGTCATCTGAATAG; CBD domain: forward, ATATATGTCGACTGCAGCCAGTACCCCTGATG; reverse, TATATAGCGGCCGCCTATTTGTTTGAACCCACATCTTC; APP full-length ectodomain: forward, ATATATGTCGACTCTGGAGGTACCCACTGATGG; reverse, TATATAGCGGCCGCCTATTTGTTTGAACCCACATCTTC. For the GST-tagged Syt-1 ectodomain construct, the entire 57 amino acids long NH_2_-terminal region of Syt-1 was amplified using FP: ATATATGGATCCATGGTGAGCGAGAGTCACCATG, and RP: TATATAGCGGCCGCCTAGGCCCACGGTGGCAATG. Following amplifications, APP fragments and the Syt-1 ectodomain were subcloned in frame with the GST tag into pGEX6P-2 vector (GE Healthcare Life Sciences, Pittsburgh, PA). Human SCN2B cDNA (BC036793) was obtained from Harvard Plasmid DNA Resource Core (Harvard Medical School, Boston, MA). NH_2_-terminal GST-tagged SCN2B ectodomain construct was generated by amplifying the ectodomain coding region of SCN2B using forward primer, ATATATGGATCCATGGAGGTCACAGTACCTGCC and reverse primer, TATATAGCGGCCGCCTAGGCCACCGTGGAGTCC and then ligated in frame with GST tag into pGEX6P-2 vector. For generation of COOH-terminal V5-tag Syt-1, Syt-2 and Syt-9 constructs, the entire coding region of Syt-1, Syt-2 and Syt-9 were amplified using the following forward and reverse primers; Syt-1: forward, ATATATGCTAGCATGGTGAGCGAGAGTCACCATG; reverse, TATATAGCGGCCGCCCTTCTTGACGGCCAGCATG; Syt-2: forward, ATATATGCTAGCATGGCAAGGAACATTTTCAAGAGGAACCAG; reverse, TATATAGCGGCCGCCCTTGTTCTTGCCCAGGAGTGC; Syt-9: forward, ATATATGCTAGCATGGCATTCCCGGAGCCCCCAAC; reverse, TATATAGAATTCGGGGCGCAGGCAGCAGC. The amplified fragments were then subcloned into pCDNA6-V5 vector (Life Technologies, Grand Island, NY). All the generated constructs were sequenced and verified at the MGH DNA sequencing core facility (Boston, MA).

### Generation and purification of GST-tagged recombinant proteins

GST-tagged ectodomain fragments of APP and SCN2B were generated and purified using similar methodology as described earlier [38]. Briefly, plasmids were transformed into *E. coli* BL21 cells (Life Technologies, Grand Island, NY) and then allowed to grow at 37 °C until the optical density of the culture reached between 0.6-1.0. Cultures were induced by 1 mM IPTG for 3 h at 24 °C. Cells were lysed and the GST-tagged recombinant proteins were purified by incubating the soluble fractions with glutathione resin for 4 h at 4 °C. Samples were washed and stored at 4 °C until further use.

### Glutathione S-transferase (GST) pull-down assay and Colloidal Blue staining

GST pull-down assays were performed as described previously [38]. In brief, adult male ICR (CD-1) mice were purchased from Charles River Laboratory and anaesthetized with the help of Isoflurane (Hospira Inc, IL). Brains were removed quickly, homogenized in the ice-cold lysis buffer containing 50 mM HEPES, pH 7.4, 100 mM NaCl, 2 mM EDTA, 1 % Triton X-100 supplemented with protease and phosphatase inhibitors cocktails (Roche Life Science, Indianapolis, IN). After removal of the insoluble fractions, soluble supernatant was incubated at 4 °C with equal amount of GST-tagged recombinant purified proteins coupled with glutathione resin. Samples were washed, eluted out and separated on one-dimensional gel electrophoresis using 4-12 % Bis-Tris Gel (Life technologies, Grand Island, NY). Gels were then subjected to Colloidal Blue staining and the excised bands were subjected to mass spectrometry-based analysis.

### Protein sequence analysis by LC-MS/MS

Excised Colloidal Blue-stained gel bands were cut into approximately 1 mm^3^ pieces and then subjected to a modified in-gel trypsin digestion procedure as described previously [39]. Gel pieces were washed, dehydrated with acetonitrile and then rehydrated with 50 mM NH_4_HCO_3_ containing 12.5 ng/μl modified sequencing-grade trypsin (Promega, Madison, WI) for 45 min at 4 °C. Peptides were extracted by removing the NH_4_HCO_3_ solution, followed by one wash with a solution containing 50 % acetonitrile and 1 % formic acid, dried and stored at 4 °C. On the day of analysis, samples were reconstituted in HPLC solvent A (2.5 % acetonitrile, 0.1 % formic acid) and loaded onto a nano-scale reverse-phase HPLC capillary column via a Famos auto sampler (LC Packings, San Francisco, CA). Peptides were eluted with the help of increasing concentrations of solvent B (97.5 % acetonitrile, 0.1 % formic acid), subjected to electrospray ionization and then entered into an LTQ Velos ion-trap mass spectrometer (Thermo Fisher, San Jose, CA). Peptides were detected, isolated, and fragmented to generate a tandem mass spectrum of specific fragment ions for each peptide. Peptide sequences (and hence protein identity) were determined by matching protein databases with the acquired fragmentation pattern by the software program Sequest (ThermoFisher, San Jose, CA).

### Immunogold electron microscopy

PC12 cells were washed and fixed in a solution containing 4 % paraformaldehyde and 0.2 % glutaraldehyde in 1X PBS. Following 5 washes, cells were pelleted, resuspended in warm 2 % agarose, cut into small blocks and incubated with 2.3 M sucrose at 4 °C for overnight. Ultrathin cryosections were generated on a Leica EM FCS at −80 °C and collected on the formvar-carbon coated nickel grids. For double immunolabeling, grids were first blocked on drops of 1 % BSA and 5 % goat serum and then incubated with mouse anti-Syt-1 antibody for 1 h followed by anti-mouse secondary antibody coupled with 10 nm gold particle for 1 h. After rinsing, grids were incubated again with rabbit anti-APP antibody for 1 h followed by anti-rabbit secondary antibody coupled with 15 nm gold particles. Grids were washed, stained on drops of Tylose and Uranyl acetate and then allowed to dry. The grids were examined at 80 kV in a JEOL JEM 1011 transmission electron microscope and the images were acquired using an AMT digital imaging system (Advanced Microscopy Techniques, Danvers, MA).

### *In situ* proximity ligation assay (PLA)

*In situ* proximity ligation assay was performed using the *In situ* PLA kit (OLink Bioscience, Sweden) according to the manufacturer’s protocol. Briefly, PC12 cells were first blocked and then incubated with rabbit anti-APP (C66) and mouse anti-Syt-1 antibody for 2 h. Cells were washed 3 times and then incubated with two different *in situ* probes for 1 h at 37 °C. After 3 washes, ligation solution was added to the cells for 30 min followed by polymerase solution for 2 h. Later, cells were mounted in the mounting medium and visualized under confocal microscope using 20X objective. Image were captured at identical settings and later processed by Metamorph software.

### Generation of Syt-1 and Syt-9 stable cell lines

Syt-1 and Syt-9 stable cell lines were generated on both CHO-APP [37] and PC12 cells. In brief, CHO-APP and PC12 cells were transfected with 4 μg of V5-tagged Syt-1 or Syt-9 cDNA with the help of Effectene transfection reagent (Qiagen, Valencia). Cells were later trypsinized and then replated in the presence of 10 μg/μl of Blasticidin selection marker (Life Technologies, Grand Island, NY). Cells were grown for 2 weeks before Blasticidin-resistant cells were further plated in a series of serial dilution in a 96 well tissue culture plate. Single cell colonies were picked and analyzed by Western blotting for optimal expression of Syt-1 or Syt-9 with the help of a mouse anti-V5 antibody.

### Primary dissociated neuronal culture

Primary dissociated neuronal culture was prepared as described previously [37].

### Lentiviral infection

Syt-1 shRNA and control DsRed lentiviral particles were generated at MGH Vector Core facility (Charlestown, MA). Mouse primary neuronal cultures were infected with 1 × 10^6^ lentiviral particles at DIV5 and half the media was replaced 12 h after infection. Cultures were allowed to grow for 10 additional days after infection before analysis of APP processing and Aβ_40_/_42_ release.

### Immunofluorescence microscopy

Primary neuronal cultures grown on cover glass in 12 well tissue culture plates were fixed at 12 days *in vitro* (DIV12) with 4 % paraformaldehyde for 30 min. Cultures were washed and permeabilized with 0.1 % Triton X-100 and 5 % donkey serum for 1 h at room temperature. Cells on coverglass were then incubated with rabbit anti-APP (C66) and mouse anti-Syt-1 antibodies at 4 °C for overnight. Following washing, cells were again incubated with Alexa Fluor488-conjugated anti-rabbit and Alexa568-conjugated anti-mouse secondary antibodies for 2 h at room temperature. Cells on coverglass were then washed and then mounted on glass slides with the help of DAPI containing mounting media (Life Technologies, Grand Island, NY). Images were acquired on Olympus IX-70 microscope at similar exposure settings and later processed by IP lab software.

### Western blot analysis

Cells were lysed in 1X GTIP buffer (10 mM Tris–HCl, pH 6.8, 150 mM NaCl, 2 mM EDTA, 1 % Triton X-100 and 0.25 % Nonidet P-40) supplemented with protease and phosphatase inhibitors cocktail. The soluble fraction was obtained by centrifugation and the protein concentration was measured with the help of a BCA protein assay kit (Pierce Biotechnology, Rockford, USA). 40–60 μg of protein samples were separated by gel electrophoresis using 4-12 % Bis-Tris gels and later transferred on PVDF membranes (Bio-Rad, Hercules, CA). Membranes were blocked with 5 % skimmed milk for 1 h at room temperature and then incubated with the indicated primary antibodies overnight at 4 °C at the following dilutions: rabbit anti-APP (C66) 1:1000, mouse anti-V5 1:5000, mouse anti-Syt-1 1:1000, rabbit anti Syt-9 1:1000, rabbit anti-sAPPβ 1:250, mouse anti-sAPPα 1:1000. Following incubation with HRP-conjugated secondary antibodies, blots were developed with the help of ECL chemiluminiscence detection reagent using Biomax film (Kodak).

### Aβ ELISA

CHO cells and PC12 cells stably expressing Syt-1 or Syt-9 and PC12 Syt-1 stable KD cells were plated on 60-mm tissue culture plates. Conditioned media was collected after 24 h and subjected to standard sandwich ELISA for measurement of secreted Aβ_40_ and Aβ_42_ using an Aβ ELISA Human/Rat kit (Wako Pure chemical). Aβ_40_ and Aβ_42_ from mouse primary neuronal cultures were determined by performing sandwich ELISA on conditioned media using 21F12/2G3 as capture antibody for Aβ_42_/ Aβ_40_ and 266 (Abeta13-26) as detector. Aβ values were normalized by the cellular protein amount and expressed as pg/ml/mg.

### Statistical analyses

All statistical analyses were performed using a 2-tailed Student’s *t* test. Error bars represented in graphs denote the SE.

## Results

### Unbiased proteomic analysis of APP ectodomain-interacting proteins

**Fig. 1:**
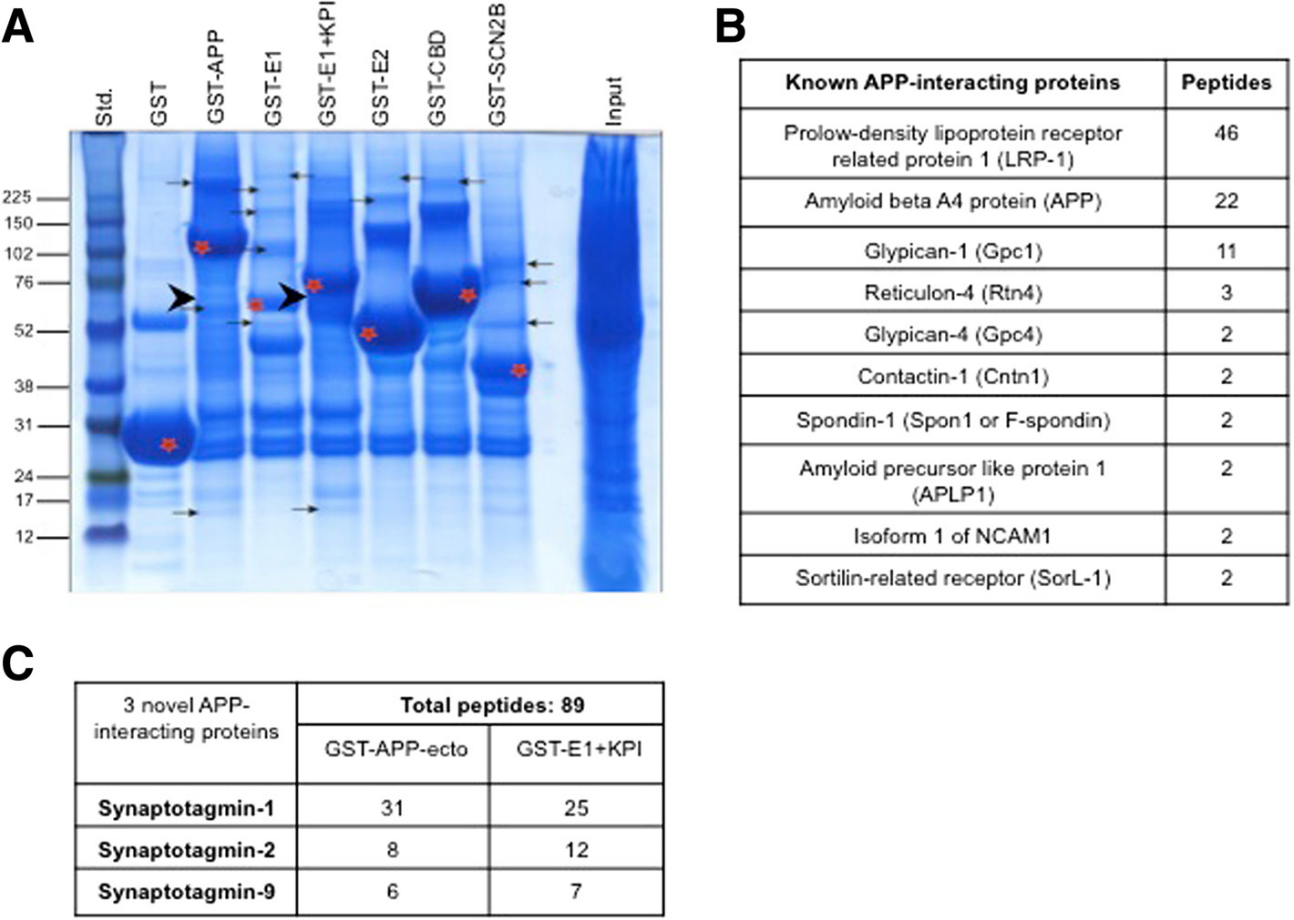
Mass spectrometry-based identification of novel APP-interacting proteins. **a** Colloidal Blue-stained gel of APP-interacting proteins from mouse forebrain extracts. The proteins were pulled down with GST-tagged APP-ectodomain regions. Red asterisks denote purified GST-tagged APP-ectodomain fragments while arrows point to the bands that were excised and subjected to MS-based analysis for protein identification. Arrowheads denote bands containing Syt peptides. **b** MS-based confirmation of previously identified APP ectodomain-interacting proteins. **c** MS-based identification of Syt-1, Syt-2 and Syt-9 peptides. Peptides from all three Syts were pulled down by GST-APP and GST-E1 + KPI. Other GST-tagged APP fragments did not pull down Syt peptides (not shown)

Proteins that regulate Aβ generation at the synapse are largely unknown. In an effort to identify these proteins, we employed an unbiased mass spectrometry-based proteomic screen using GST-tagged APP ectodomain regions as baits. The five APP ectodomain protein fragments used in these experiments are listed in Additional file 1. Figure S1A: full ectodomain of APP (GST-APP), E1 domain (GST-E1), E1 and KPI domains together (GST-E1 + KPI), E2 domain (GST-E2), and E2 and carbohydrate-binding domains together (GST-CBD). Immobilized GST-tagged recombinant proteins were used to pull-down interacting proteins from adult mouse forebrains extracted in 1 % Triton X-100 lysis buffer. The interacting protein complexes were separated by one-dimensional gel electrophoresis, stained with Colloidal Blue stain and the excised bands were subjected to mass spectrometry analysis (Harvard Taplin MS facility). As shown in Fig. 1a, each GST-tagged APP ectodomain fragment pulled down distinctive sets of protein bands indicative of multiple interacting proteins. Moreover, these Colloidal Blue-stained bands were not visible in the GST tag alone fraction suggesting specific association of these proteins with the APP domains and not with the GST tag.

Mass spectrometry analysis of the Colloidal Blue-stained bands revealed numerous proteins in each fraction. As shown in Fig. 1b, our unbiased screen successfully identified a large number of peptides belonging to proteins already known to interact with the APP ectodomain [40–43], validating the specificity of our proteomic screen. In addition to the known APP ectodomain-interacting proteins, we have found multiple novel proteins as well. Based on the number of peptides and spectra, our screen identified Synaptotagmin-1, −2, and −9 as major APP ectodomain binding proteins (Fig. 1c). Syts account for a total of 89 peptides in ~50-70 kDa bands (Fig. 1c). The number of Syt peptides identified is in the order of peptides that were detected for multiple known APP ectodomain-interacting proteins in our unbiased assay, suggesting that the Syt family of proteins is a potentially strong APP-interacting protein family. All three Syts were found in both the GST-APP (full-length ectodomain) and the GST-E1 + KPI fractions. Other fractions of APP, GST alone, or the GST-SCN2B ectodomain did not contain any Syt peptides, confirming the specificity of our assay. Since the E1 domain alone did not pull down Syts, our data suggest that this protein family preferentially interacts with the KPI domain or the region surrounding the KPI domain. Altogether, these data suggest that Syts are novel APP-interacting proteins.

### APP interacts with Syt-1, Syt-2 and Syt-9

**Fig. 2:**
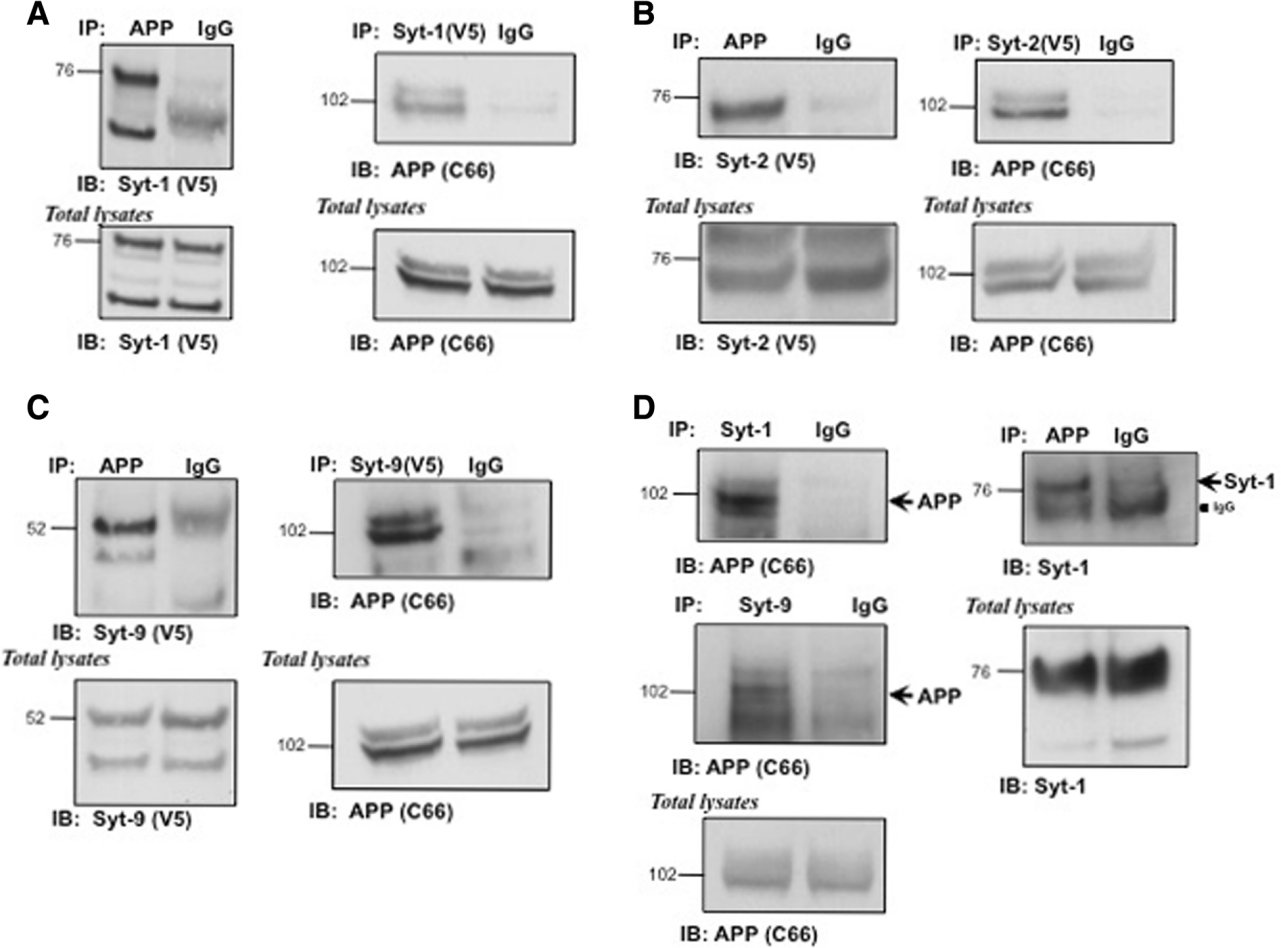
APP interacts with Syt-1, −2 and −9 *in vitro* and *in vivo.* **a** Western blot analysis of APP and Syt-1 co-immunoprecipitates. CHO cells stably coexpressing APP and V5-tagged Syt-1 were subjected to immunoprecipitation using specific APP (C66) or V5-tag antibodies. Syt-1 or APP specific bands were identified in the immunoprecipitates but were not observed in the control IgG pull-down (*n* = 3 for each condition). **b** Co-immunoprecipitation of APP and Syt-2. APP or Syt-2 were immunoprecipitated from CHO cells expressing Syt-2 (V5-tag) and probed with anti-V5 or APP (C66) antibodies. APP specific bands were identified in the fraction immunoprecipitated with anti V5 antibody (Syt-2) while Syt-2 specific bands were identified in the APP pull-down (*n* = 3 for each condition). **c** Western blot analysis showing co-immunoprecipitation of APP with Syt-9. CHO cells stably co-expressing APP and V5-tagged Syt-9 were subjected to immunoprecipitation using specific APP (C66) or V5-tag antibodies. Syt-9 or APP specific bands were identified in the immunoprecipitates but were not observed in the control IgG pull-down (*n* = 3 for each condition). **d** Western blot analysis of APP and Syt-1 or Syt-9 co-immunoprecipitates from adult mouse forebrain. Specific antibodies against endogenous Syt-1 or Syt-9 were used to immunoprecipitate Syt-1 or Syt-9 and probed with an anti-APP antibody. Western blot shows APP-specific staining in both Syt-1 and Syt-9 immunoprecipitates as compared to the IgG controls. In a reverse co-immunoprecipitation assay, the APP-specific antibody co-immunoprecipiated Syt-1 (*n* = 2 for each condition)

To directly assess whether Syt-1, Syt-2 and Syt-9 interact with APP, we performed co-immunoprecipitation experiments. CHO cells stably co-expressing APP and Syt-1, Syt-2, or Syt-9 were lysed and immunoprecipiated with anti-APP C66 antibodies. As shown in Fig. 2, APP antibodies successfully pulled down bands around ~ 50–70 kDa specific for Syt-1, Syt-2, and Syt-9 (Figs. 2a, b, and c respectively). In a reverse co-immunoprecipitation assay, anti-V5 antibodies against V5-tagged Syts were also able to pull down both mature and immature forms of APP from stably expressing CHO cells (Figs. 2a, b, and c). The association of APP with different forms of Syt family members was specific, as neither APP nor Syt-1, −2 or −9 were significantly detected in the control IP fraction with IgG (Figs. 2a, b, and c). This data suggest that APP interacts with all three isoforms of Syts in cells.

Next, we performed co-immunoprecipitation assays from adult mouse forebrains to test whether Syt-APP complexes also exist *in vivo*. Adult mouse forebrains were extracted with 1 % Triton X-100 and incubated with either anti-APP C66 antibodies or mouse anti-Syt antibodies along with the appropriate control IgG antibodies. As shown in Fig. 2d, both mature and immature forms of APP were pulled down with Syt-1 and Syt-9 specific antibodies but not with control IgG antibodies from adult mouse forebrains. Conversely, anti-APP antibodies also co-immunoprecipitated Syt-1 from forebrains (Fig. 2d). As Syt-2 specific antibodies are not commercially available, we were not able to test whether APP also exists in complex with Syt-2 *in vivo*. Taken together, these data clearly demonstrate that all three Syts form complexes with APP *in vitro* and Syt-1 and −9 *in vivo,* and further validate our initial unbiased proteomic screen results that Syt-1, −2 and −9 are novel APP-interacting proteins.

### Syt-1 is a physiological interactor of APP

**Fig. 3:**
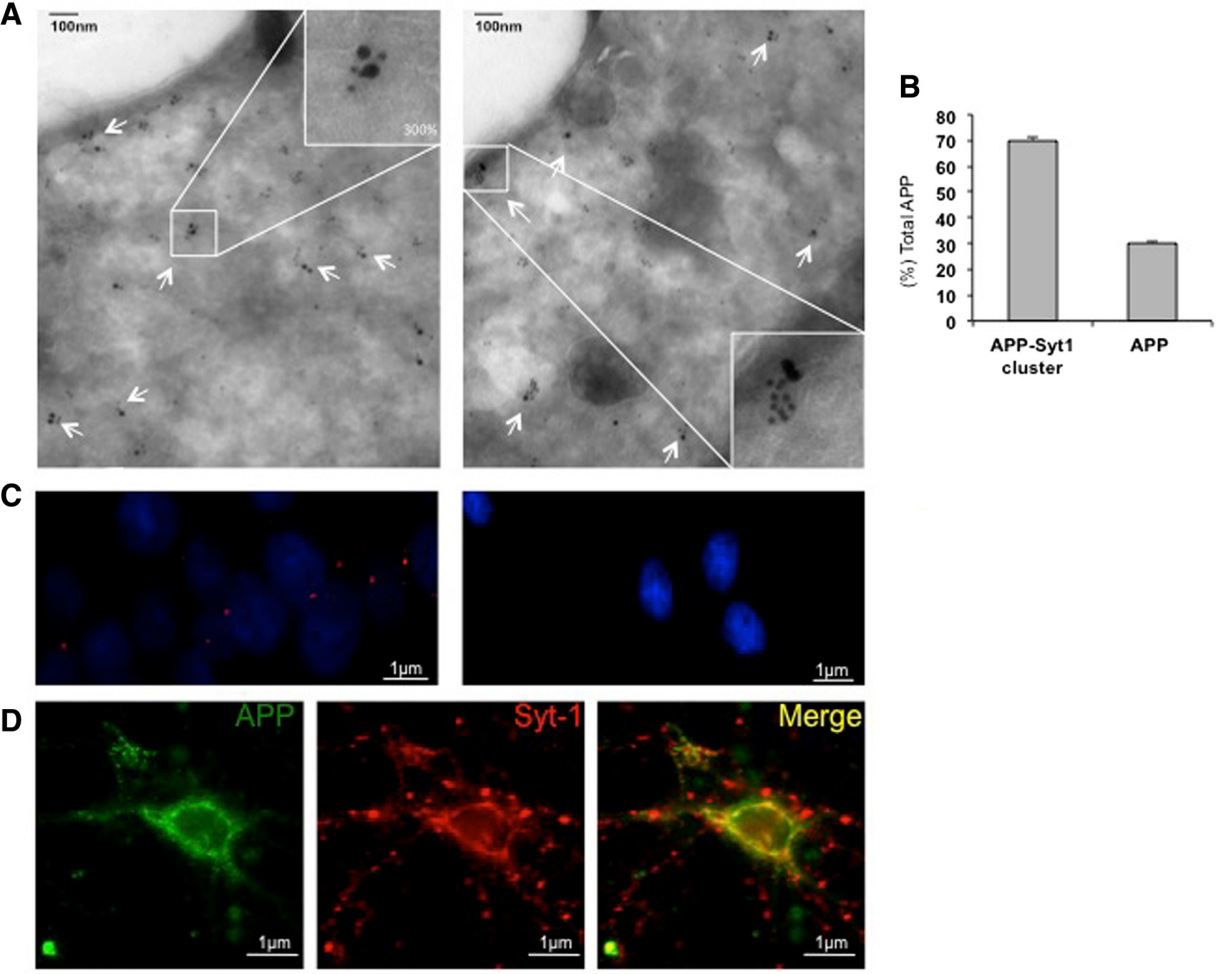
Syt-1 is a physiological interactor of APP. **a** Electron microscopic (EM) images of ultra-thin cryosections of naïve PC12 cells show double immunogold labeling of endogenous APP (15 nm) with Syt-1 (10 nm). White arrows depict co-localization of APP with Syt-1 while the inserts show 300 % magnification of APP in close association with Syt-1. Analysis was based on three independent sets of experiments. **b** Quantitative analysis of immunogold labeling of APP in cluster with Syt-1 compared to APP alone, as revealed by **a. c** *In situ* PLA shows interaction between endogenous APP and Syt-1 in naïve PC12 cells. Fluorescence red dots represent close association between APP and Syt-1 (left panel) as compared to the negative control (right panel). Images were taken at identical settings. Nuclear staining with DAPI is shown in blue. Analysis was based on three independent sets of experiments. **d** Immunofluorescence microscopy images of mouse primary neuronal cultures stained with APP and Syt-1 specific antibodies. Co-localization between APP (green) and Syt-1 (red) was observed in the cell body and in neurites. Images were obtained at identical settings. Analysis was based on three independent sets of experiments

In addition to co-immunoprecipiation assays, we also performed image-based analysis experiments to detect physiological interaction between endogenous APP and Syt-1. PC12 cells derived from rat adrenal glands have previously been used as a well-defined model to study Syt-1 protein function. In PC12 cells, Syt-1 is known to reside in large dense core vesicles (LDCVs) that resemble synaptic vesicle morphology in neuronal cells [44, 45]. We next performed electron microscopy (EM) analysis to detect interaction between endogenous APP and Syt-1 proteins residing in PC12 cell vesicles. PC12 cells were fixed and ultrathin cryosections were subjected to double-immunogold labeling using specific primary antibodies against APP and Syt-1. As shown in Fig. 3a, immunogold labeling revealed a potential oligomerization pattern of Syt-1 by itself. Interestingly, the majority of the large gold particle-labeled APP protein was observed in close proximity with the cluster of Syt-1 proteins (Fig. 3a, insert). Quantitative analysis revealed that ~70 % of APP was found clustered with Syt-1 with a distance between the two gold particles of less than 30 nm (Fig. 3b). This indicates robust co-localization between the two proteins.

We further validated the interaction between APP and Syt-1 at an endogenous level by an *in situ* proximity ligation assay (PLA) and immunofluorescence analysis. *In situ* PLA was performed in PC12 cells. PC12 cells were fixed and then subjected to Duolink *in situ* PLA using anti-APP (C66) and anti-Syt-1 antibodies. Co-localization of Syt-1 and APP antibodies together in intact PC12 cells resulted in a significant increase in polymerization and ligation of *in situ* probes. These were visualized as fluorescent red dots, indicating direct interaction between Syt-1 and APP in PC12 cells (Fig. 3c left panel). On the other hand, no fluorescent red dots were observed in the negative control when PC12 cells were labeled with only one primary antibody either against APP or Syt-1 (Fig. 3c right panel). Immunofluorescence analyses were carried out in primary neuronal cultures. Dissociated primary neuronal cultures at DIV12 were fixed, permeabilized and subjected to APP and Syt-1 labeling together using specific antibodies. As shown in Fig. 3d, robust colocalization of APP (green) with Syt-1 (red) was observed in the neuronal cell body as well as in the neurites. Altogether, these data show that APP interacts with Syt-1 at an endogenous level both in PC12 cells and in primary neurons.

### Syts interact with the linker sequence between the E1 and KPI domains of APP

Our unbiased APP ectodomain-interacting protein screen identified multiple peptides that belong to three different members of Syt family of proteins. Other than the GST-APP full ectodomain fraction, multiple Syt peptides were also found in the GST-E1 + KPI domain fraction but not in any other GST fractions. These initial results suggested that the KPI domain of APP or the 108-amino acids region between the E1 and KPI domains represent the primary site of Syt interaction with the APP ectodomain.

**Fig. 4:**
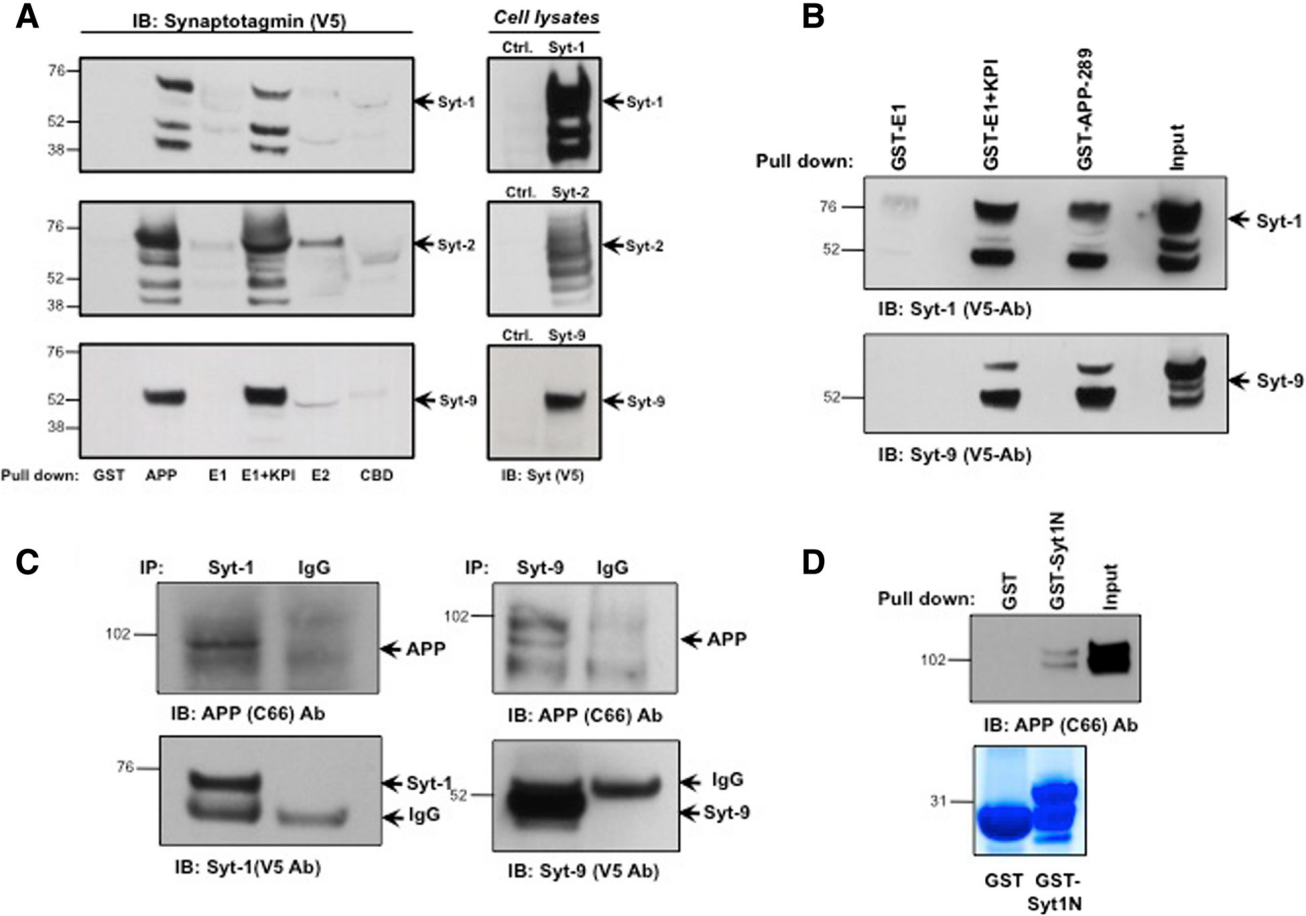
APP interacts with Syt-1 and Syt-9 via its linker region between the E1 and KPI domain. **a** *In vitro* GST pull-down assay of Syt-1, −2 and −9. GST-tagged APP-ectodomain fragments were used to pull down V5-tagged Syt-1, −2 and −9 from stably expressing CHO cells. Western blot analysis with anti-V5 antibodies revealed specific interaction of Syt-1, −2 and −9 with both GST-APP-ectodomain and GST-E1 + KPI fragments. Analysis was based on 4 different sets of experiments. **b** *In vitro* GST pull-down assay showing interaction of Syt-1 and Syt-9 with the linker region of APP between the E1 and KPI domain. The GST-tagged APP-289 fragment (containing the linker region but not the KPI domain) pulled down both Syt-1 and Syt-9 while the GST-E1 domain did not. Analysis was based on three different sets of experiments. **c** Co-immunoprecipitation of the APP_695_ isoform with Syt-1 and Syt-9. CHO cells stably expressing APP_695_ were transfected with V5-tagged Syt-1 or Syt-9. Western blot analysis shows specific co-immunoprecipitation of APP_695_ with V5-tagged Syt-1 and Syt-9 (*n* = 3 for each condition). **d** *In vitro* GST pull-down assay showing that the Syt-1 N-terminal region interacts with APP. Purified GST-Syt1 N-terminal region pulled down full-length APP while no interaction was observed with control GST. Bottom panel shows separate colloidal blue stained gel of purified GST and GST-Syt1 N-terminal purified proteins used for the APP pull-down assay. Analysis was based on four different sets of experiments

To confirm this initial result, we employed a direct *in vitro* GST pull-down assay. CHO cells were transiently transfected with V5-tagged Syt-1, −2, or −9 isoforms and exposed to the immobilized GST-APP ectodomain fragments. Western blot analysis revealed that GST-APP pulled down two or more bands for all three Syts (Fig. 4). The presence of two or more Syt bands is likely due to known glycosylation-dependent post-translational modifications of these proteins [32]. All forms of all three Syts were specifically pulled down with the GST-tagged APP ectodomain and GST-E1 + KPI fragments but not the other APP ectodomain fragments (Fig. 4a). We have not excluded that APP may prefer one form of Syt over another. A very weak nonspecific interaction of Syt-2 was also observed with the E2 domain of APP. These data indicate that the KPI domain and/or the region between the E1 and KPI domains serve as the primary site of Syt interaction on APP.

Next, we asked whether the KPI domain or the region between the E1 and KPI domains mediate the interaction of APP with Syts. For this purpose, we first engineered a stop codon at the beginning of the KPI domain and then generated the purified APP ectodomain fragment (GST-APP289) that contains the E1 domain and the 108 amino acids linker between the E1 and KPI domain but not the KPI domain. CHO cells stably expressing either V5-tagged Syt-1 or Syt-9 were lysed and the cell lysate was then exposed to various immobilized GST-tagged APP ectodomain fragments. The GST-APP289 region, but not the GST-E1 or GST alone fragments, was able to pull down Syt-1 and Syt-9 from CHO cells (Fig. 4b).

To directly demonstrate that the KPI domain is not essential in mediating APP-Syt-1/Syt-9 interaction, we performed co-immunoprecipitation experiments using the APP_695_ isoform that lacks the KPI domain. CHO cells stably transfected with APP_695_ were transiently transfected with either V5-tagged Syt-1 or Syt-9 and the cell extracts were immunoprecipitated using anti-V5 antibodies. Syt-1 and Syt-9 were able to pull-down both mature and immature form of the APP_695_ isoform as compared to the control IgG, confirming that the KPI domain is dispensable for APP and Syt-1/Syt-9 interaction (Fig. 4c). These data show that the linker region between the E1 and KPI domain, and not the KPI domain, mediates the interaction of APP with Syts.

As Syt-1 interacts with APP ectodomain region, we next tested whether the APP interaction site lies in the lumenal N-terminal region of Syt-1. Similarly to the APP ectodomain constructs, the entire Syt-1 N-terminal lumenal region of 57 amino acids was GST-tagged, expressed, purified and used as a bait to pull-down APP in an *in vitro* GST pull-down assay (Additional file 1: Figure S1B). As shown in the Fig. 4d, APP was pulled down with the Syt-1 N-terminal fragment while no binding was observed with the control GST tag alone. This suggests that Syt-1 and APP most likely interact through their lumenal N-terminal ectodomain regions.

### Syt-1 and Syt-9 regulate APP processing and Aβ generation

**Fig. 5:**
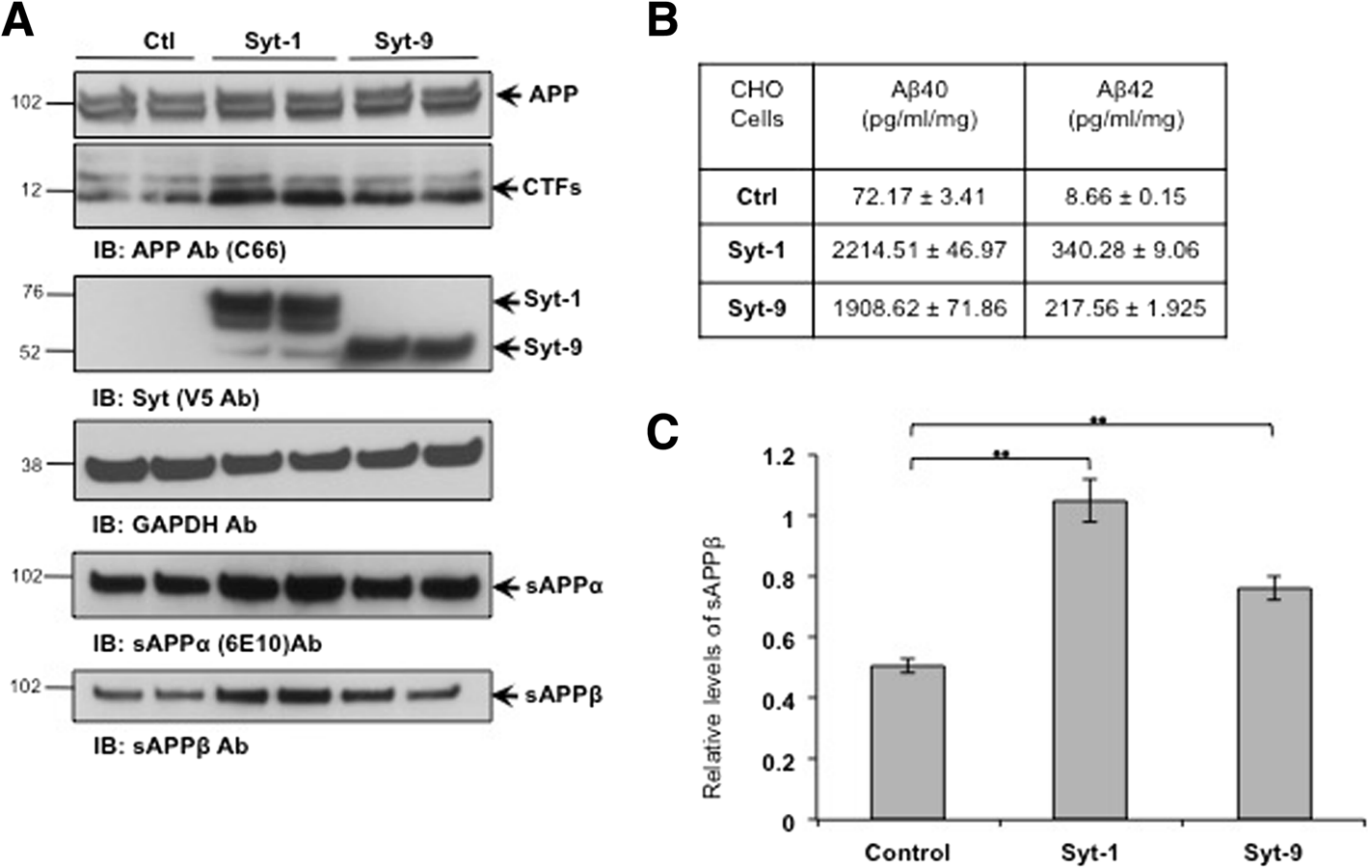
Syt-1 and Syt-9 modulate APP processing and Aβ levels in CHO cells. **a** Western blot analysis of CHO cells stably co-expressing Syt-1 or Syt-9 with APP shows a significant increase in APP-CTFs, sAPPα and sAPPβ levels as compared to the control CHO-APP cells. Syt-1 and Syt-9 expression was confirmed using anti-V5 tag antibodies and a GAPDH antibody was used for equal protein loading. **b** Sandwich ELISA from the conditioned media indicates a strong increase in Aβ_40_ and Aβ_42_ release from cells expressing Syt-1 or Syt-9 as compared to the control CHO-APP cells. **c** Quantitative analysis of total sAPPβ levels in the conditioned media of CHO cells co-expressing Syt-1 or Syt-9 with APP as compared to only APP expression (student *t* test; **, p < 0.01; *n* = 4 for each condition)

As both Syt-1 and Syt-9 interact with APP in cells and in mouse brains, we next tested the effect of Syt-1 and Syt-9 expression on APP metabolism and Aβ generation. Syt-1 or Syt-9 were stably co-expressed with APP in CHO cells and the cell lysate was subjected to Western blot analysis using an anti-APP antibody (C66). Stable overexpression of either Syt-1 or Syt-9 in CHO cells significantly increased APP-CTFs levels as compared to only APP-expressing cells while no changes were observed in mature or immature APP levels (Fig. 5a).

As overexpression of Syts regulate APP processing in CHO cells, we next analyzed the effect of Syt-1 or Syt-9 expression on Aβ levels in the same system. Conditioned media from CHO cells stably co-expressing Syt-1 or Syt-9 with APP was subjected to Aβ_40_ and Aβ_42_ ELISAs as described in the methods section. Stable co-expression of Syt-1 with APP caused a ~30-fold increase in Aβ_40_ levels and a ~39-fold increase in Aβ_42_ while a ~25-fold increase in Aβ_40_ and Aβ_42_ levels was observed in cells expressing Syt-9 with APP (Fig. 5b).

Stable over expression of Syt-1 or Syt-9 enhanced APP processing as seen by elevated APP-C83 and APP-C99 levels, suggesting that Syt-1 and Syt-9 affect both α- and β-cleavage of APP. To test this, we also analyzed sAPPα and sAPPβ levels in the conditioned media of CHO cells expressing Syt-1 or Syt-9 with APP. Fig. 5a and c show significantly elevated levels of both sAPPα and sAPPβ in cells expressing either Syt-1 or Syt-9 as compared to APP-expressing cells. Together, our results indicate that expression of Syt-1 and Syt-9 in CHO cells markedly elevates APP-CTFs, sAPP and Aβ levels.

### Syt-1 and Syt-9 regulate endogenous APP-CTF and Aβ levels in PC12 cells

**Fig. 6:**
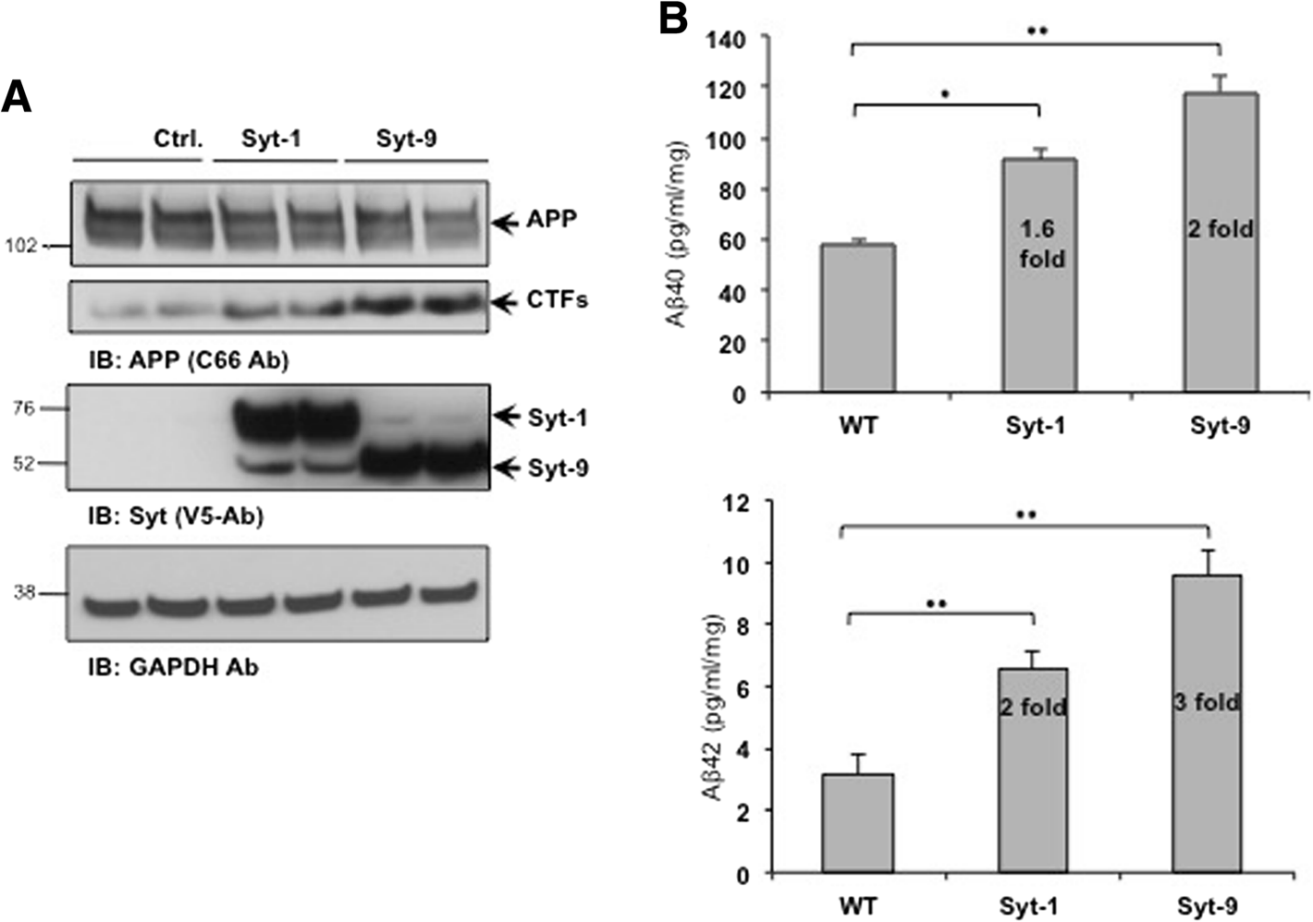
Expression of Syt-1 and Syt-9 in PC12 cells increases endogenous APP-CTF and Aβ levels. **a** Western blot analysis shows that stable expression of Syt-1 or Syt-9 in PC12 cells increases endogenous APP-CTF levels. Bottom panel shows expression of Syt-1 and Syt-9 and a GAPDH antibody was used for equal protein loading. **b** Quantitative analysis of sandwich ELISA from the conditioned media of PC12 cells co-expressing Syt-1 or Syt-9 with APP. Expression of Syt-1 or Syt-9 in PC12 cells causes a robust increase in secreted Aβ_40_ and Aβ_42_ levels as compared to the control APP-expressing cells (student *t* test; *, p < 0.05; **, p < 0.01; *n* = 4 for each condition)

While expression of Syt-1 or Syt-9 dramatically increases APP-CTF and Aβ levels in CHO cells expressing APP, CHO cells do not exhibit neuronal characteristics. Thus, we next tested the effect of Syt-1 and Syt-9 expression on endogenous APP processing in PC12 cells. PC12 cells stably expressing either Syt-1 or Syt-9 were lysed and the lysates were subjected to Western blot analysis using the anti-APP (C66) antibody. No significant changes in full-length APP levels were observed with either Syt-1 or Syt-9 expression in PC12 cells. However, both Syt-1 and Syt-9 significantly elevated endogenous APP-CTF levels in PC12 cells as compared to the parental cells (Fig. 6a). Endogenous APP-C99 could not be clearly distinguished from APP-C83 in PC12 cells.

In addition to APP-CTFs, we also analyzed the effect of Syt-1 or Syt-9 expression on endogenous Aβ levels. Conditioned media collected from the PC12 cells expressing Syt-1 and Syt-9 were subjected to Aβ_40_ and Aβ_42_ ELISAs. A ~ 2- to 3-fold increase in Aβ_40_ and Aβ_42_ levels were observed with the expression of Syt-1 or Syt-9 in PC12 cells as compared to parental cells (Fig. 6b). Taken together, data obtained from both CHO-APP cells and native PC12 cells show that expression of Syt-1 and Syt-9 increases endogenous APP-CTF and Aβ_40_ and Aβ_42_ levels.

### Syt-1 knockdown reduces APP-CTF, sAPPβ and Aβ levels

**Fig. 7:**
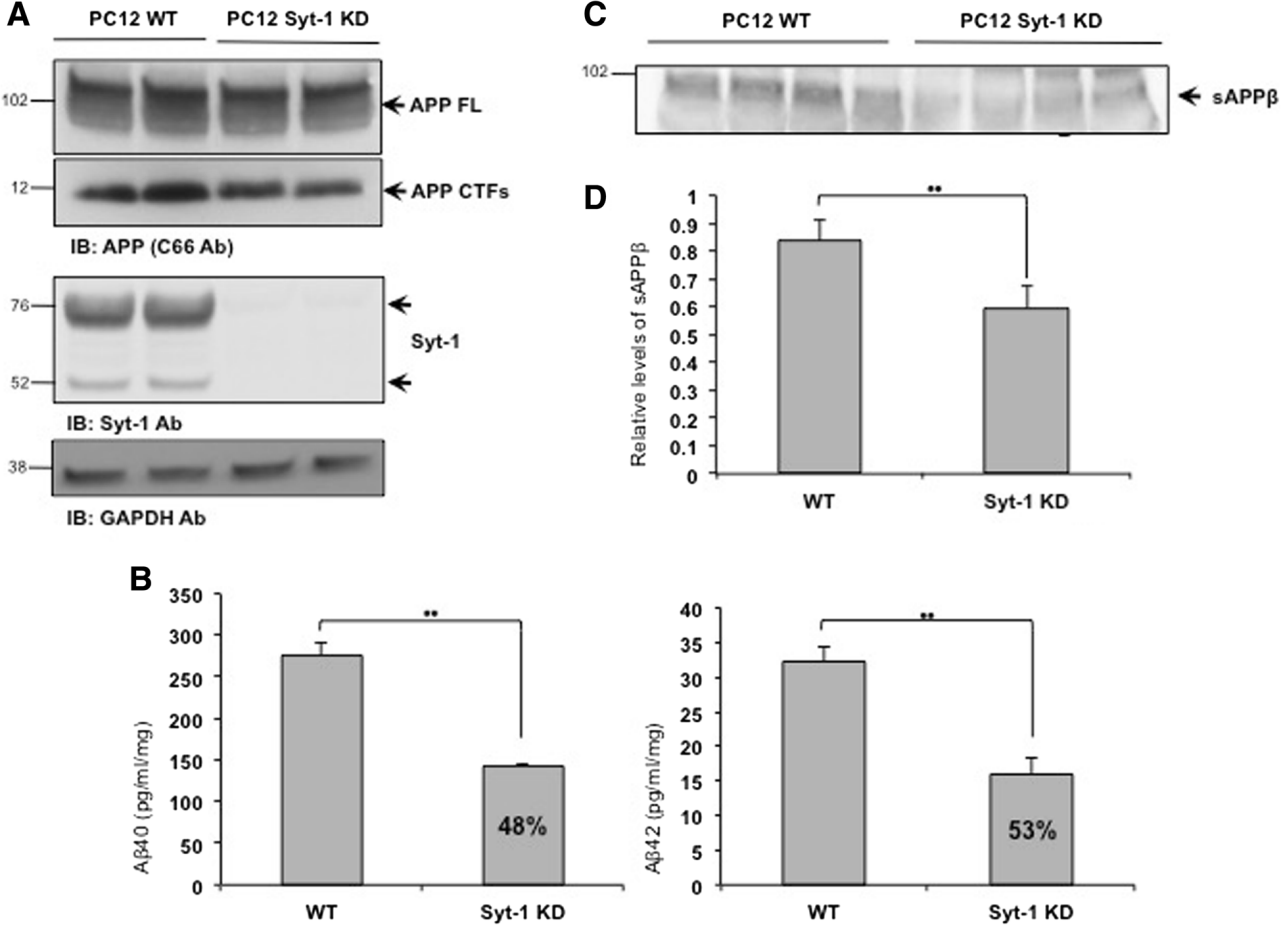
Stable knockdown of Syt-1 reduces endogenous APP-CTF, Aβ and sAPPβ levels in PC12 cells. **a** Stable knockdown of Syt-1 in PC12 cells reduces endogenous APP-CTF levels as revealed by Western blot analysis. Knock down of Syt-1 was confirmed with a Syt-1 specific antibody (Synaptic Systems) and a GAPDH antibody was used for equal protein loading. **b** Quantitative analysis of sandwich ELISA shows lower Aβ_40_ and Aβ_42_ levels in the conditioned media of the PC12 cells with stable Syt-1 KD as compared to the WT cells (student *t* test; **, p < 0.01; *n* = 3 for each condition). **c** Western blot analysis of conditioned media shows a significant reduction in total sAPPβ levels in stable Syt-1 KD PC12 cells as compared to the WT cells. **d** Quantitative analysis of total sAPPβ levels in the conditioned media of the WT PC12 cells and stable Syt-1 KD PC12 cells (student *t* test; **, p < 0.01; *n* = 3 for each condition)

In order to assess the role of endogenous Syt-1 expression in the regulation of APP metabolism, we next used a knockdown approach to lower the expression of endogenous Syt-1 and analyze its effect on APP metabolism and Aβ levels. PC12 cells that stably express shRNA against Syt-1 were generated and characterized as described earlier [46, 47]. Wild type (WT) and Syt-1 knockdown (KD) PC12 cells were plated and the cell lysates were subjected to Western blotting with an anti-APP (C66) antibody. A significant reduction in APP-CTF levels was observed in Syt-1 KD PC12 as compared to wild type cells, while no changes were noted in full-length APP levels (Fig. 7a).

In addition to stable expression of shRNA against Syt-1, we also employed a siRNA-based strategy to transiently knock down the endogenous expression of Syt-1 and confirm a decrease in APP-CTF levels. A 90-95 % reduction in endogenous Syt-1 expression was observed, as revealed by Syt-1 specific antibody. Transient expression of Syt-1 specific siRNAs resulted in significant reduction of endogenous APP-CTF levels in PC12 cells, without changing full-length APP levels (Additional file 1: Figure S2).

We also measured secreted Aβ_40_ and Aβ_42_ levels in PC12 cells with reduced Syt-1 expression. Conditioned media from both WT and stable Syt-1 KD PC12 cells were collected and subjected to Aβ_40_ and Aβ_42_ ELISAs. Decreased Syt-1 expression resulted in a 48 % reduction in secreted Aβ_40_ levels with a concomitant 53 % decrease in Aβ_42_ levels, as compared to the WT PC12 cells (Fig. 7b).

Lack of Syt-1 expression in PC12 cells lowers endogenous APP-CTF generation and secreted Aβ_40_ and Aβ_42_ levels, suggesting that Syt-1 may regulate BACE1-mediated cleavage of APP. To test this, we analyzed secreted sAPPβ in WT versus Syt-1 KD PC12 cells. Figures 7c and d show that stable Syt-1 KD PC12 cells release ~ 30 % less sAPPβ in the conditioned media as compared to the WT PC12 cells. All together, these results indicate that endogenous Syt-1 promotes BACE1-mediated cleavage of endogenous APP in PC12 cells.

### Syt-1 regulates Aβ levels in mouse neurons

**Fig. 8:**
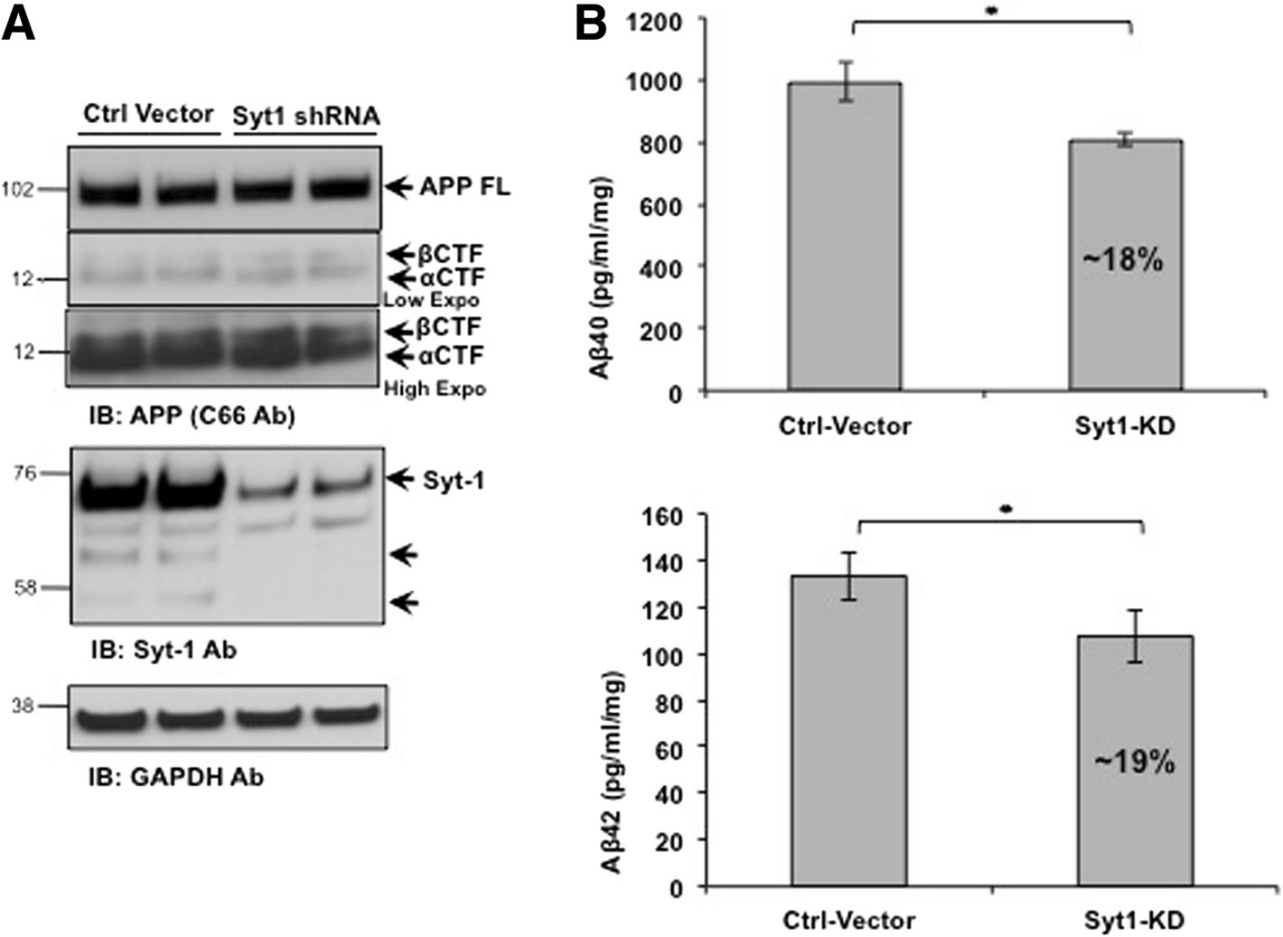
Lentiviral-mediated knock down of Syt-1 reduces endogenous Aβ levels in mouse primary neuronal culture. **a** Western blot analysis of APP CTF levels in mouse primary neurons infected with lentiviral shRNA against Syt-1. Reduction in the endogenous expression of Syt-1 was confirmed using a Syt-1 specific antibody (Synaptic Systems) while GAPDH staining was used to ensure equal protein loading. **b** Quantitative analysis of Aβ_40_ and Aβ_42_ levels from the conditioned media of mouse primary neurons infected with a control vector and lentiviral vector expressing Syt-1 shRNA. Lentiviral-mediated knock down of endogenous Syt-1 in mouse primary neuronal cultures significantly decreases secreted Aβ_40_ and Aβ_42_ levels as compared to the control vector (student *t* test; *, p < 0.05; *n* = 3 for each condition)

Using both over expression and knock down strategies, we have clearly shown that Syt-1 is an important regulator of APP processing and Aβ generation in PC12 cells. To study if Syt-1 modulates APP processing and Aβ generation in mouse primary neurons, we employed a lentiviral-mediated approach to knock down endogenous Syt-1 expression. A lentiviral vector that expresses shRNA against mouse Syt-1 was used to knock down Syt-1 expression along with an empty control vector. Mouse primary neuronal cultures were infected with lentiviral vectors at DIV5 and the cultures were maintained for 10 additional days before analysis for APP CTFs levels and Aβ_40_/Aβ_42_ generation. As shown in the Fig. 8a, lentiviral-mediated infection of mouse primary neuronal cultures led to ~70 % reduction in Syt-1 expression levels as compared to the control. Interestingly, when we analyzed the conditioned media from the infected cultures, we observed a significant 18-19 % reduction in secreted Aβ_40_ and Aβ_42_ levels with Syt-1 knock down as compared to the controls (Fig. 8b). The continued expression of low levels of Syt-1 and other Syts in these cultures may have compensated for the loss of function of Syt-1 in the neuronal system as compared to the PC12 cells. Indeed, we had observed interaction between APP and other members of the Syt family. Thus, a ~20 % reduction in Aβ_40_ and Aβ_42_ levels in the mouse primary culture system is expected. All together, our data show that Syt-1 is an endogenous regulator of Aβ generation in mouse primary neurons.

## Discussion

APP regulates numerous physiological functions in brain, but it is also cleaved to generate Aβ species involved in the pathology of the Alzheimer’s disease [48–50]. To understand how APP functions are regulated in both physiological and pathological conditions, numerous studies have focused on the identification of APP-interacting proteins [28, 29, 51]. Although these studies identified some mouse brain APP-associated proteins, these mainly bound to the APP-CTF. Only a few proteins such as contactin, F-spondin and Nogo-66 have been reported so far to interact with the APP extracellular domains [41, 52, 53].

Here, we performed an unbiased proteomic screen using purified GST-tagged APP-ectodomain fragments and have identified members of the Synaptotagmin family as novel APP-ectodomain interacting proteins. Using transgenic mice, previous studies had identified Syt-1 and Syt-11 as pulling down with APP and thus suggested a close association between these Syts and APP [29, 54]. However, these studies have not characterized the interaction between APP and Syts and its effect on APP processing. Our data not only identify Syt-1 as an APP-interacting protein but also show that Syt-1, −2, and −9 exist in complex with APP both *in vitro* and *in vivo*, the interaction is mediated by APP’s linker region between the E1 and KPI domains, and it results in altered APP processing.

The 17 different isoforms of Synaptotagmins not only differ in structure but also in subcellular localization [55, 56]. Syt-1 and Syt-2 were shown to localize to synaptic vesicles while Syt-9 is targeted to both axons and dendrites [57]. Recently, it was shown that APP is localized to synaptic vesicles and trafficked via neuronal activity-dependent release of synaptic vesicles [36]. The interaction of Syts with APP could mediate the localization of APP to synaptic vesicles and its activity-dependent localization to presynaptic membranes. Syt-1 is the best-characterized isoform among the Synaptotagmin family of proteins. Syt-1 acts as a dual sensor as it regulates both the fusion of synaptic vesicles to the pre-synaptic membrane as well as their endocytosis [58]. Endocytosis has been shown to play an important role in the generation of Aβ as APP is endocytosed via a clathrin-mediated process from the plasma membrane into endosome-like structures to generate Aβ peptides [59, 60]. These studies suggest the possibility that Syt-1 containing vesicles may acquire APP during synaptic vesicle endocytosis from the presynaptic membrane and regulate APP processing and Aβ generation in the endosomes. However, the precise role of Syt-1 in APP trafficking, localization, and metabolism warrants further investigation.

APP is known to localize both at the presynaptic and postsynaptic terminals where it acts as a trans synaptic adhesion molecule and promotes synapse formation [18]. Presence of APP at the synapse regulates synaptic transmission and neurotransmitter release, best shown in APP/APLP2 double knockout mouse models. Indeed, APP/APLP2 double knockout mice exhibit reduced synaptic vesicle density, active zone size, and number of docked synaptic vesicles per active zone as compared to APLP2 single knockout [21, 61, 62]. Moreover, overexpression of APP in PC12 cells promoted exocytosis and increased basal and constitutive secretion [63]. Given the essential role of Syt-1 in synaptic vesicle exocytosis, docking and neurotransmitter release [30], its interaction with APP may regulate its function. This could partially or fully explain altered synaptic vesicle biology in APP knockout mice. Future studies will be needed to fully explore the role of APP in regulating Syt-1-mediated synaptic vesicle exocytosis and endocytosis.

Evidence suggests that APP undergoes BACE1 and PS1/γ-secretase-dependent proteolytic processing in the presynaptic compartments where it may generate Aβ peptides released from the nerve terminals [25]. Genetic inactivation of PS1 in the presynaptic compartment significantly increases APP-CTF accumulation, supporting a role for the presynaptic compartment as a major site for APP processing and Aβ generation [64]. In addition, PS1 and other γ-secretase complex components were found in purified synaptic vesicle preparations from adult rat brain [35]. Similarly to the γ-secretase complex components, BACE1 was also found in the synaptic vesicle fraction [36, 65]. Moreover, using a transgenic AD mouse model, Vassar *et al.* have observed strong colocalization of BACE1 with APP in the swollen dystrophic presynaptic terminals surrounding Aβ plaques [66]. This suggests abnormal accumulation of BACE1 at presynaptic sites and enhanced BACE1-mediated processing of APP, potentially contributing to AD pathogenesis. Our results indicate that an APP-interacting protein, Syt-1, regulates APP processing in the presynaptic compartment. Specifically, our data suggest that Syt-1 modulates BACE1-mediated cleavage of APP as overexpression of Syt-1 enhanced sAPPβ and Aβ levels while loss of Syt-1 in PC12 cells resulted in lower endogenous Aβ generation and sAPPβ levels in the conditioned media with decreased APP-CTF levels. Moreover, our results indicate that Syt-1 also regulates α-cleavage of APP and loss of Syt-1 may also impair α-cleavage as we have observed increased sAPPα in Syt-1 overexpressing cells. BACE1-mediated cleavage of APP in presynaptic vesicles was previously reported [65]. Interestingly, the pH of synaptic vesicles is around 5.0 - 5.7, which would allow for an ideal microenvironment for optimal BACE1 activity and Aβ generation [67, 68]. Given the essential role of Syt-1 in synaptic vesicle biology and trafficking, it is very likely that BACE1-mediated cleavage of APP and Aβ generation are regulated by Syt-1 particularly in synaptic vesicles. Thus, Syt-1 interaction with APP could represent a novel therapeutic target in the treatment or prevention of AD.

## Additional file

Additional file 1: Figure S1. Schematic representation of GST-tagged APP and Syt-1 ectodomain. (S1A) Schematic diagram of APP structure *(top)* and GST-tagged APP ectodomain *(bottom)* constructs used in MS-based identification of APP-interacting proteome from mouse brain. (S1B) Schematic representation of Syt-1 structure *(top)* and GST-tagged Syt-1 N-terminal (lumenal region) construct. **Figure S2**. Syt-1 siRNA decreases endogenous APP-CTF levels in PC12 cells. Western blot analysis of PC12 cells transiently transfected with Syt-1 specific siRNA shows lower APP-CTF levels as compared to the control cells. Top panel shows full-length APP and APP-CTF levels while bottom panel shows Syt-1 expression. GAPDH staining was used for equal protein loading.
