# Supplementary material for: Synaptotagmins interact with APP and promote Aβ generation
Source: Mol Neurodegener. 2015 Jul 23;10:31. doi: 10.1186/s13024-015-0028-5 (PMC4511450; doi:10.1186/s13024-015-0028-5)
Supplement: Additional file 1: Figure S1. — Schematic representation of GST-tagged APP and Syt-1 ectodomain. (S1A) Schematic diagram of APP structure (top) and GST-tagged APP ectodomain (bottom) constructs used in MS-based identification of APP-interacting proteome from mouse brain. (S1B) Schematic representation of Syt-1 structure (top) and GST-tagged Syt-1 N-terminal (lumenal region) construct. Figure S2. Syt-1 siRNA decreases endogenous APP-CTF levels in PC12 cells. Western blot analysis of PC12 cells transiently transfected with Syt-1 specific siRNA shows lower APP-CTF levels as compared to the control cells. Top panel shows full-length APP and APP-CTF levels while bottom panel shows Syt-1 expression. GAPDH staining was used for equal protein loading. [file 13024_2015_28_MOESM1_ESM.pdf]

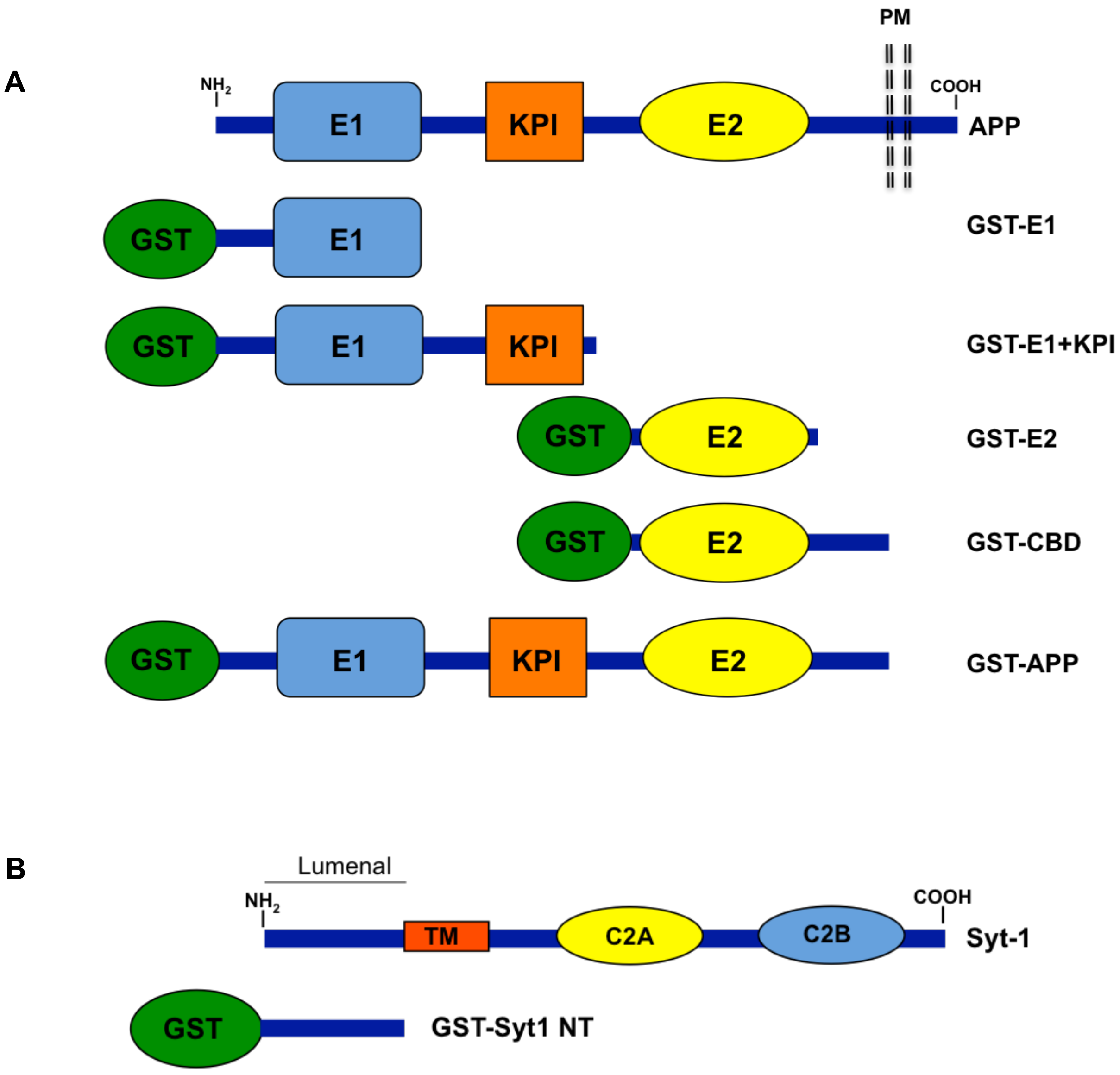

Fig S1. Schematic representation of GST-tagged APP and Syt-1 ectodomain

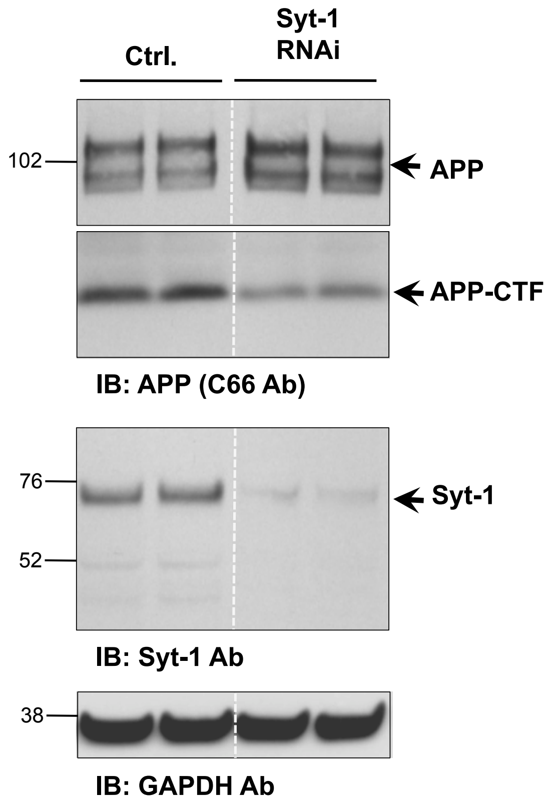

Fig S2. Syt-1 siRNA decreases endogenous APP CTF levels in PC12 cells

Supplementary Figure Legends

Figure S1. Schematic representation of GST-tagged APP and Syt-1 ectodomain

(S1A) Schematic diagram of APP structure *(top)* and GST-tagged APP ectodomain *(bottom)* constructs used in MS-based identification of APP-interacting proteome from mouse brain.

(S1B) Schematic representation of Syt-1 structure *(top)* and GST-tagged Syt-1 N-terminal (lumenal region) construct.

Figure S2. Syt-1 siRNA decreases endogenous APP-CTF levels in PC12 cells

Western blot analysis of PC12 cells transiently transfected with Syt-1 specific siRNA shows lower APP-CTF levels as compared to the control cells. Top panel shows full-length APP and APP-CTF levels while bottom panel shows Syt-1 expression. GAPDH staining was used for equal protein loading.
